# Targeted Delivery of Salusin‐α Into Rabbit Carotid Arterial Endothelium Using SonoVue

**DOI:** 10.1002/jum.15714

**Published:** 2021-04-05

**Authors:** Yuxue Wang, Min Luo, Xiaolu Mao, Xiaoyan Shi, Xiang Liu

**Affiliations:** ^1^ Department of Laboratory Medicine Hubei University of Chinese Medicine Wuhan China; ^2^ Central Laboratory, The Central Hospital of Wuhan, Tongji Medical College Huazhong University of Science and Technology Wuhan China; ^3^ Key Laboratory for Molecular Diagnosis of Hubei Province, The Central Hospital of Wuhan, Tongji Medical College Huazhong University of Science and Technology Wuhan China

**Keywords:** carotid arterial endothelium, plasmids, Salusin‐α, SonoVue, targeted delivery

## Abstract

**Objectives:**

A new method based on the adhesion of SonoVue to plasmids was assessed to achieve targeted gene delivery into the vascular endothelium.

**Methods:**

pEGFP‐Salusin‐α and pcDNA3.1‐Salusin‐α plasmids were transfected into the arterial endothelium of different rabbit groups. Western blotting was performed to analyze the expression of EGFP and salusin‐α in the common carotid arteries of rabbits from different groups, and ELISA was performed to detect plasma salusin‐α levels in rabbits from each group; simultaneously, blood parameters of different groups of rabbits were measured.

**Results:**

Green fluorescence was observed in the right common carotid artery of rabbits transfected with pEGFP‐Salusin‐α, but not in the endothelial cells of not‐transfected control rabbits. The expression of salusin‐α in the transfected animals was higher than that in the control not‐transfected animals (*P* < .05). In rabbits transfected with pcDNA3.1‐Salusin‐α plasmid, salusin‐α expression was higher than in the not‐transfected control animals (*P* < .05). However, there was no significant difference in plasma salusin‐α levels between transfected animals and controls (*P* > .05). Blood parameters were also measured in both groups.

**Conclusions:**

Our data confirm the establishment of a new method using SonoVue for targeted gene delivery into the arterial endothelium. Our study outcomes propose a new method of intervention in atherosclerosis and a new tool for targeted gene delivery.

**Abbreviations**
ALB: albumin
ALT: alanine aminotransferase
AST: aspartate aminotransferase
BUN: blood urea nitrogen
CEUS: contrast‐enhanced ultrasonography
CK: creatine kinase
CREA: creatinine
EGFP: enhanced green fluorescent protein
GLU: glucose
HB: hemoglobin
HCT: hematocrit
LDH: lactate dehydrogenase
MI: mechanical index
PBS: phosphate‐buffered saline
PCR: polymerase chain reaction
PLT: platelet count
RBC: red blood cell count
TP: total protein
WBC: white blood cell count

Cardiovascular disease carries one of the highest mortality rates worldwide,^1^, ^2^ especially due to atherosclerosis.^3^, ^4^ Atherosclerosis is usually characterized by the proliferation of blood vessel walls, plaque formation, tissue necrosis, and eventually the rupture of blood vessels, leading to thrombosis.^5^, ^6^, ^7^ Preventing the progression of atherosclerosis is often challenging and there is currently no simple cure available. Gene therapy might be a promising effective method to treat atherosclerosis in the future.^8^, ^9^, ^10^ Research on pathogenic mechanisms of atherosclerosis has provided many target genes for gene therapy,^11^, ^12^ which could be the basis for effective clinical intervention in atherosclerosis. Gene therapy involves the introduction of normal exogenous genes into a patient's target cells to treat a disease caused by a defect or abnormality of a specific endogenous gene. However, gene therapy involves the inherent technical challenges of transferring selected therapeutic genes into patients' target cells.

SonoVue is an ultrasound diagnostic contrast agent that is commonly used in clinics. Its working principle exploits the potential of its active substance sulfur hexafluoride to form microbubbles that can enhance the backscattered echo signals of ultrasonic instruments and significantly augment perfusion imaging of normal and diseased tissues, in order to improve the discrimination, sensitivity, and specificity of ultrasonic diagnosis.^13^, ^14^ Studies on the relationship between ultrasound and contrast microbubbles showed that increasing the intensity of ultrasound could cause microbubbles to burst quickly.^15^, ^16^ Insonation of microbubbles could produce liquid jets and local shear stress, which would alter biological membranes and facilitate gene transport into cells. Moreover, in our previous papers, we observed that, on the basis of this interaction, plasmids incorporated inside ultrasound contrast agent microbubbles were efficiently transported into the endothelium of the common carotid artery of rabbits.^17^, ^18^ However, it had not been investigated whether the use of SonoVue microvesicles could point‐direct plasmid delivery into the endothelium. The present study introduces a new method of targeted gene transfer to achieve expression of the vasoactive peptide salusin‐α into the endothelial cells of rabbit carotid artery, which could be in the near future applied for clinical gene therapy.

## Materials and Methods

### Preparation of Plasmid and SonoVue Mixture

Construction of pEGFP‐Salusin*‐α* and pcDNA3.1‐Salusin*‐α*.

Human salusin*‐α* sequence (84‐bp), synthesized by Shanghai Biotechnology, was inserted between the XhoI and BamHI digestion sites, into pEGFP‐N1 vector (Invitrogen Life Technologies, Carlsbad, CA, USA) to generate pEGFP‐salusin*‐α*.^17^ Similarly, the salusin*‐α* sequence was cloned into pcDNA3.1 (Invitrogen Life Technologies) using the same two digestion sites and pcDNA3.1‐Salusin*‐α* was generated. The polymerase chain reaction (PCR) forward primer sequence was.

5′‐TGTGGGATCCGGTGCCCTTCCTCCCGCT‐3′ and the PCR reverse primer sequence was 5′‐TATACTCGAGTCCCTTGGCTCCAGGCCC‐3′.

The two plasmids were sequenced to verify the success of molecular cloning. High‐purity plasmids were prepared on a large scale for use in the experiments described below (Invitrogen DNA Purification Kit. No. 12280096A).

Based on our previous study,^17^ we prepared the mixture in the ratio of 20 μL SonoVue (45 μg/mL) solution and 1 μL plasmid (1 μg/μL) solution and tested the adhesion rates of different plasmids and SonoVue, as described below.

### SonoVue Adherence Ratio to Plasmids

To evaluate the adherence ratio of SonoVue, varying amounts of SonoVue (45 mg/mL), which were prepared according to the manufacturer's instructions, were mixed with the same amount of plasmids (1 mg/mL). The total volume was the same after 0.9% sodium chloride (NaCl) solution was added to each mixture. The mixture was incubated for 15 minutes at room temperature and separated into two layers. The upper layer had a very thin white layer, which contained SonoVue adhered to plasmid molecules. The lower layer was a thick solution including free plasmids not included in SonoVue microbubbles. After incubation, ultraviolet spectrophotometry was performed to test plasmid concentration in the lower layer; absorbance (*A*) of each mixture was determined by measuring the optical density at 260 nm (*A*_260_) and 280 nm (*A*_280_) and using the following equation:

$$\text{Plasmid concentration}\;\left(\mathrm{\mu g}/\mathrm{mL}\right)=\left(A_{260}-A_{280}\right)\;\times \text{the dilution factor}\;33$$

(from the Cold Spring Harbor online protocol, the plasmid concentration and adherence ratio were calculated)

$$\text{SonoVue adherence ratio}\;=\left(1-\text{Down}-\text{layer plasmids}\;\left(\mathrm{\mu g}\right)\right)\%$$

### Rabbit Grouping and Plasmid Transfection

All experimental New Zealand white rabbits (~2.5 kg) were obtained from Hubei Medical Experimental Animal Center and treated following protocols approved by the Institute of Animal Care and Use Committee of Hubei University of Chinese Medicine (approval document number 2015025, time: 2015‐6‐3). As described in the Guide and the Animal Welfare Act Regulations, rabbits were maintained in our laboratory under standard conditions and fed an ordinary diet every day.^17^, ^18^

In order to analyze the transfection efficiency of pEGFP‐salusin*‐α* and pcDNA3.1‐Salusin*‐α* into the arterial tunica intima, nine rabbits were divided into three groups: the pEGFP‐salusin*‐α* (pES) transfection group (*n* = 3), for which successful transfection of vascular endothelium was judged by expression of the enhanced green fluorescent protein (EGFP) gene; the pcDNA3.1‐Salusin*‐α* (pcS) transfection group (*n* = 3) where transfection efficiency was measured by checking the expression of salusin‐α; and the pH 7.4, phosphate‐buffered saline (pBS) group (*n* = 3) as the control group, where only a mixture of phosphate‐buffered saline (PBS) pH 7.4 (containing no plasmid) and SonoVue was administered.

**Figure 1:**
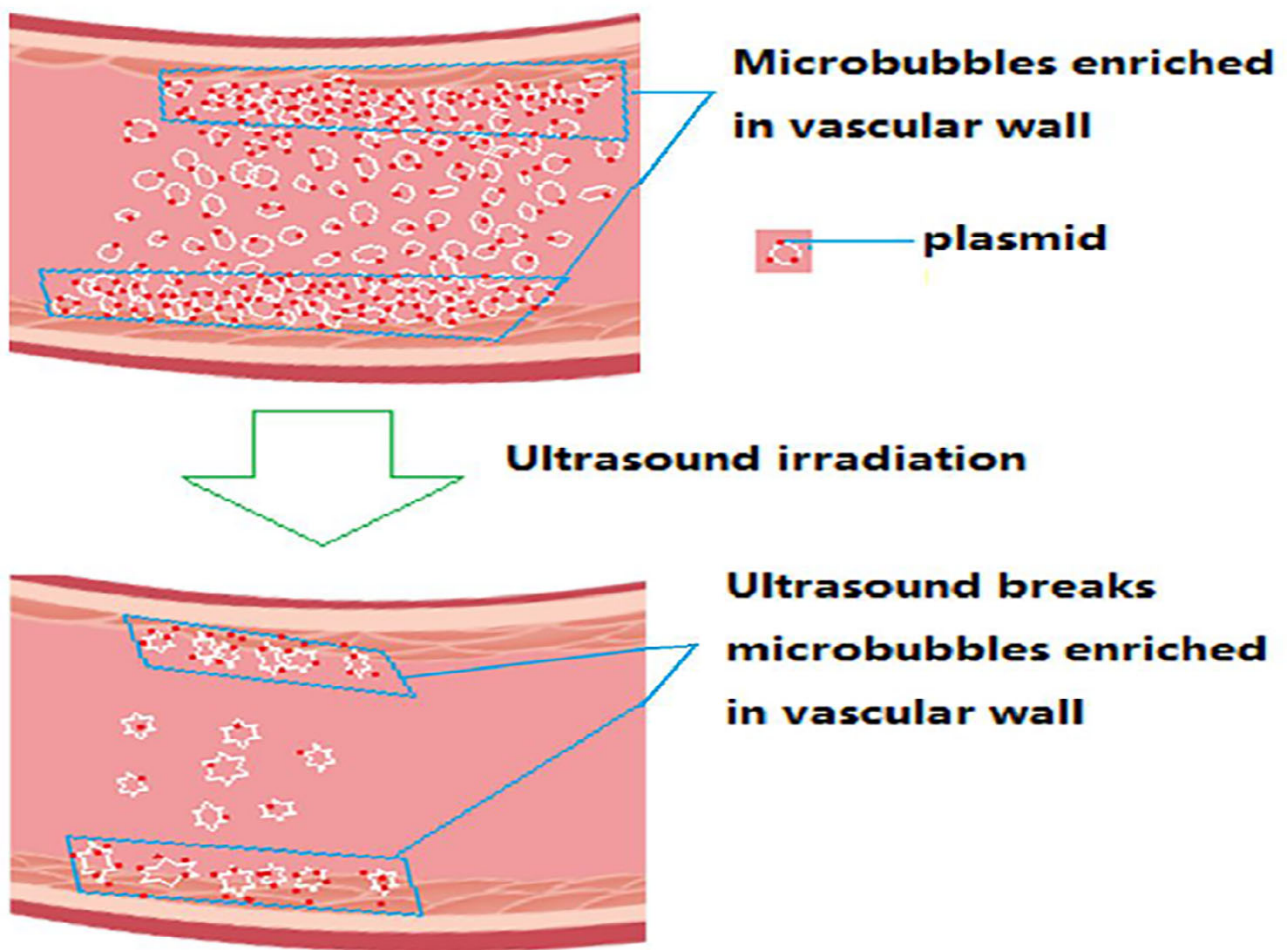
Proposed mechanism of plasmid translocation into vascular endothelium using SonoVue. This is a diagram of a possible mechanism by which SonoVue may transport plasmids into the endothelium. A mixture of SonoVue microvesicles and plasmids (which have adhered to the microvesicles) was injected into the artery and became enriched in the vascular wall. Ultrasonic irradiation ruptured the microbubbles and propelled the plasmids into the endothelium.

The plasmid transfection method was the same as that used in our original study.^17^, ^18^ In brief, a mixture of 100 μL of plasmids (1 μg/μL) and 2 mL of SonoVue (45 μg/mL) was incubated for 20 minutes at room temperature’ and then mixed and immediately injected through a 22‐gauge intravenous cannula into the ear vein of anesthetized (pentobarbital sodium 25 mg/kg; Invitrogen) rabbits. After injection, the right common carotid artery was ultrasonicated for 3 minutes until SonoVue microbubbles had been largely disrupted from the circulatory system. This transfection method is based on the hypothesis of transport mechanism of plasmid into vascular endothelium by SonoVue, shown in Figure 1. During the course of the experiment, plasmid transfection was carried out after 2 weeks with one transfection cycle being performed each week, once a week. Two weeks later, all rabbits' blood and left and right common carotid arteries were obtained. To minimize pain, rabbits were anesthetized and prevented from observing the culling process.

### Baseline and Contrast‐Enhanced Ultrasonography Examination Settings

All operating procedures were the same as described in our previous studies.^17^, ^18^ Table 1 shows the baseline and contrast‐enhanced ultrasonography examination settings of the clinical ultrasound scanner (Siemens Acuson Sequoia 512, Siemens, Germany) used in this study. The conventional carotid artery examination mode was used for baseline ultrasound examination, and the mechanical index (MI) was 1.5. Low MI real‐time contrast‐enhanced ultrasonography (CEUS) examination was carried out with contrast pulse‐sequencing imaging mode, MI was 0.18. Clinical ultrasound scanner detection data were stored on a hard disk.

**Table 1:** Ultrasound Scanner Settings

Parameter	Baseline Examination	CEUS Examination
Machine type	Siemens Acuson Sequoia 512 15L8W‐S probe	
Imaging mode	2‐D imaging	Contrast pulse sequencing imaging
MI (as read on screen)	1.5	0.18; 1.5
Frequency (MHz)	10.0–14.0	10.0–14.0
Depth (cm)	2–2.5	2–2.5
Focus position (cm)	0.5–1	0.5–1
Focus number	1	1

Parameter settings of ultrasound scanner in contrast‐enhanced ultrasonography (CEUS); MI, mechanical index.

### Rabbit Blood Parameters and Vascular Pathology

Blood was collected from all rabbits at the end of experiments and EDTA‐2K was used as anticoagulant. The following blood parameters were measured according to the laboratory blood test protocol: white blood cell count (WBC), red blood cell count (RBC), hemoglobin (HB), hematocrit (HCT), platelet count (PLT), total protein (TP), albumin (ALB), alanine aminotransferase (ALT), aspartate aminotransferase (AST), creatine kinase (CK), lactate dehydrogenase (LDH), blood urea nitrogen (BUN), creatinine (CREA), and glucose (GLU). Blood test values were compared between pBS and pcS groups.

To investigate vascular intimal hyperplasia, histopathological examination of vascular tissue sections was performed. Similar to the routine pathological examination previously described,^17^, ^18^ paraffin‐embedded blocks of carotid arterial tissue obtained from rabbits in each group were sliced into 4‐μm thick sections, stained with hematoxylin–eosin (HE), and examined by a trained pathologist. Using an Axiophot microscope (Zeiss, Austin, TX, USA) and a digital camera (Leaf Systems Lumina, Southborough, MA, USA), magnified images were captured and processed using a software from Optima Imaging Analysis Systems (Version 6.5, Media Cybernetics, Silver Springs, MD, USA).

Arterial sections from the animals in the pES group were harvested after 2 weeks with one transfection cycle being performed each week, once a week. Fast‐frozen sections were prepared for checking EGFP expression with fluorescence microscopy, and for testing salusin‐α level in arterial tissue by western blotting.

### Western Blot and ELISA

In order to analyze protein expression in the carotid arteries of the rabbits, western blotting was performed as described in our previous studies.^17^, ^18^ Carotid artery samples were homogenized in 1 mL lysis buffer (50 mM NaCl, 10 mM Tris, 1 mM EDTA, 1 mM PMSF, 0.5 mM Na_3_VO_4_·12H_2_O, 50 mM NaF, and 1 mM benzamidine), and centrifuged at 12,500*g* for 15 minutes at 4°C. Protein concentration was determined using BCA Protein Assay Reagents (Pierce Com., Rockford, IL, USA). For each sample, equal amounts of protein lysate (20 μg) were loaded onto 7.5% sodium dodecyl sulfate‐polyacrylamide gels for electrophoresis and then transferred onto polyvinylidene difluoride membranes (Amersham Pharmacia Biotech, Piscataway, NJ, USA). The membranes were blocked in PBS supplemented with 3% (w/v) bovine serum albumin for 1 hour at room temperature. The membranes were cut into strips according to protein molecular weight standard, and the strips expected to contain different molecular weight proteins were incubated overnight at 4°C with different primary antibodies, as follows: anti‐EGFP rabbit polyclonal antibodies (OpenBiosystems, Ohio, USA; cat. no. CAB4211), anti‐salusin‐α rabbit polyclonal antibodies (Yansheng, Shanghai, China; cat. no. YS‐KT2539), and anti‐glyceraldehyde‐3‐phosphate dehydrogenase (GAPDH; Santa Cruz, CA, USA; cat. no. sc‐25778). Horseradish peroxidase‐conjugated goat anti‐rabbit IgG2a antibody (Santa Cruz; cat. no. sc‐2061) was used as a secondary antibody. Western blots were visualized using enhanced chemiluminescence detection reagents (Sigma, St. Louis, MO, USA) following manufacturer's instructions. Protein bands were quantified by scanning with Bio‐Rad GelDoc XR and Chemi Doc XRS systems (Bio‐Rad, Hercules, CA, USA), and analyzed using Quantity One 1‐D Analysis Software Version 4.6.3 (Bio‐Rad).

Human salusin‐α ELISA kits were obtained from USCN Life Science Inc. (Wuhan, China). Salusin‐α standard curves were generated according to manufacturer's instructions. Before examining the samples, the best dilution of serum samples was determined. A microtiter plate provided with the kit was pre‐coated with a salusin‐α antibody. Standards or samples were added to the appropriate microtiter plate wells with a biotin‐conjugated salusin‐α polyclonal antibody and avidin‐conjugated to horseradish peroxidase (HRP), and incubated at 37°C. TMB (3, 3′, 5, 5′ tetramethyl‐benzidine) substrate solution was added to each well, and the plates were incubated at 37°C until the enzyme‐substrate mixed solution exhibited a change in color. The enzyme‐substrate reaction was terminated by the addition of a sulfuric acid solution, and the color change was measured spectrophotometrically at a wavelength of 450 nm. The concentration of salusin‐α in the samples was detected by comparing the OD values of the samples with the standard curve depending on the dilution.

### Statistical Analysis

All data are presented as mean ± standard deviation. SPSS (version 19.0; SPSS, Chicago, IL, USA) was used for statistical analysis, and the mean results of different groups were analyzed by ANOVA to determine statistical significance. Differences with *P* values of less than .05 were considered significant. All experiments were performed three times.

## Results

### Adhesion of Plasmid to SonoVue

**Figure 2:**
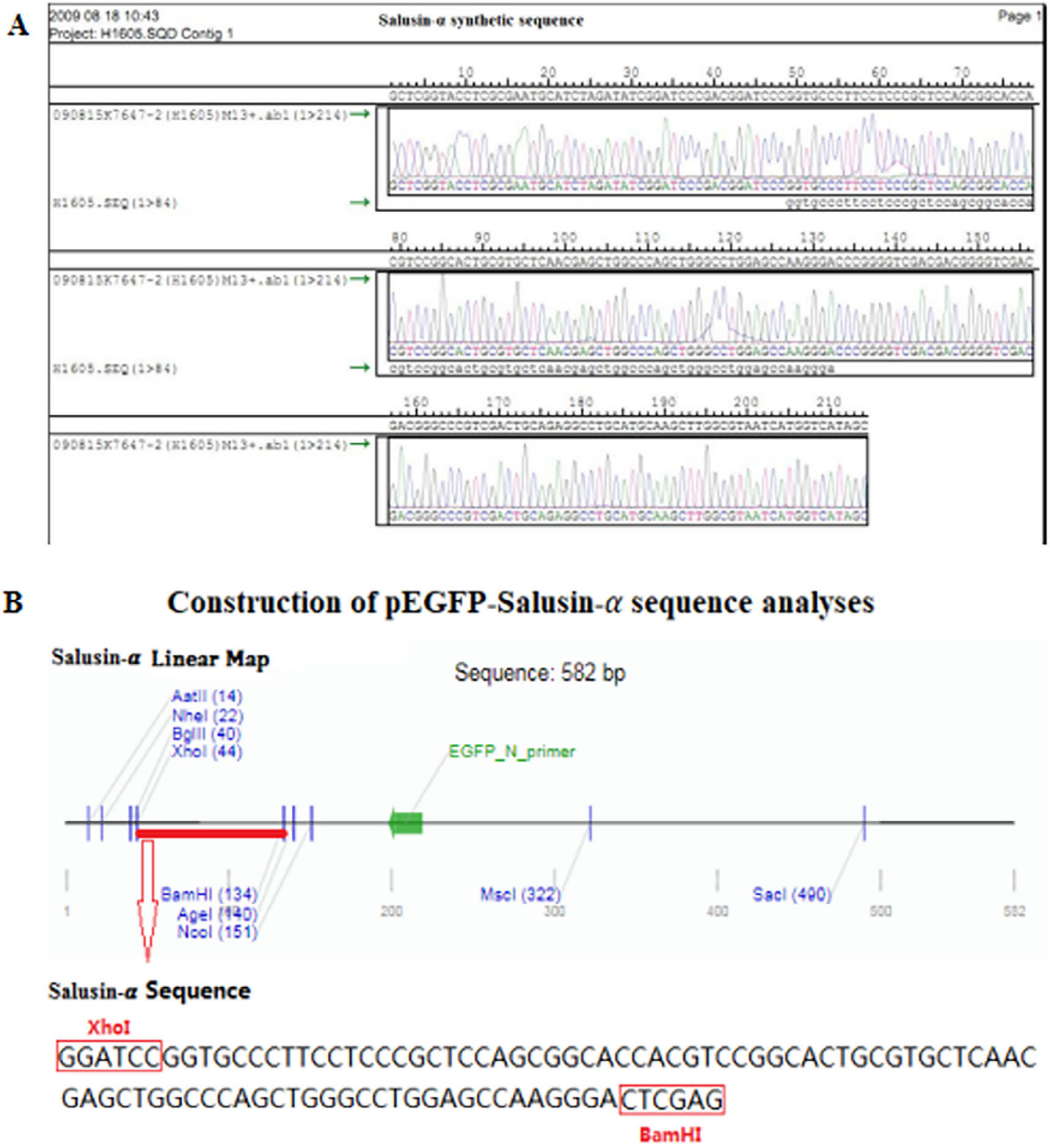
Construction of pEGFP‐Salusin‐α plasmid.

The nucleotide sequence encoding for the vasoactive peptide salusin‐α was synthesized by Shanghai Sangon, and is shown in Figure 2A. Salusin‐α sequence was amplified by PCR and cloned into pEGFP‐N1, to obtain pEGFP‐salusin‐α. pEGFP‐salusin‐α was then sequenced and its sequence was analyzed using Addgene website sequence analysis software to verify correct cloning (Figure 2B). The same was done for plasmid construct pcDNA3.1‐Salusin‐α.

**Figure 3:**
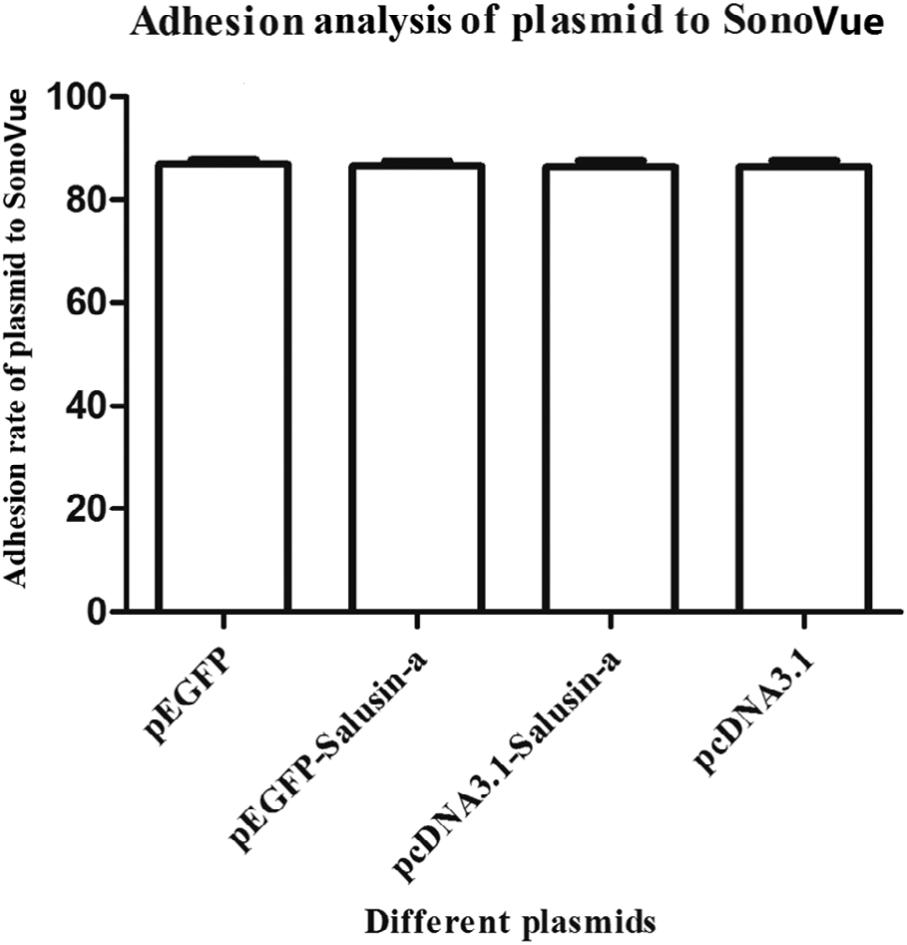
Adhesion analysis of plasmid and SonoVue. The adhesion rate of all plasmids to SonoVue was approximately 89%. There was no significant difference among SonoVue adhesion rates as measured for each of the four plasmids. The data are representative of three independent experiments (*n* = 3).

As shown in Figure 3, we performed a comparative analysis of SonoVue adhesion rates to the four plasmids: pEGFP‐N1, pEGFP‐salusin‐α, pcDNA3.1‐Salusin‐α, and pcDNA3.1. There was no difference among SonoVue adhesion rates to the four plasmids, suggesting that the sequence cloned into the plasmid did not affect SonoVue adhesion rate to the plasmid. SonoVue adhesion rate to all plasmids was 89%. In the present study, we chose pEGFP‐salusin‐α and pcDNA3.1 Salusin‐α plasmids for transfection into the vascular endothelium.

### Analysis of Rabbit Carotid Arterial Endothelium after Transfection with pEGFP‐Salusin‐α

**Figure 4:**
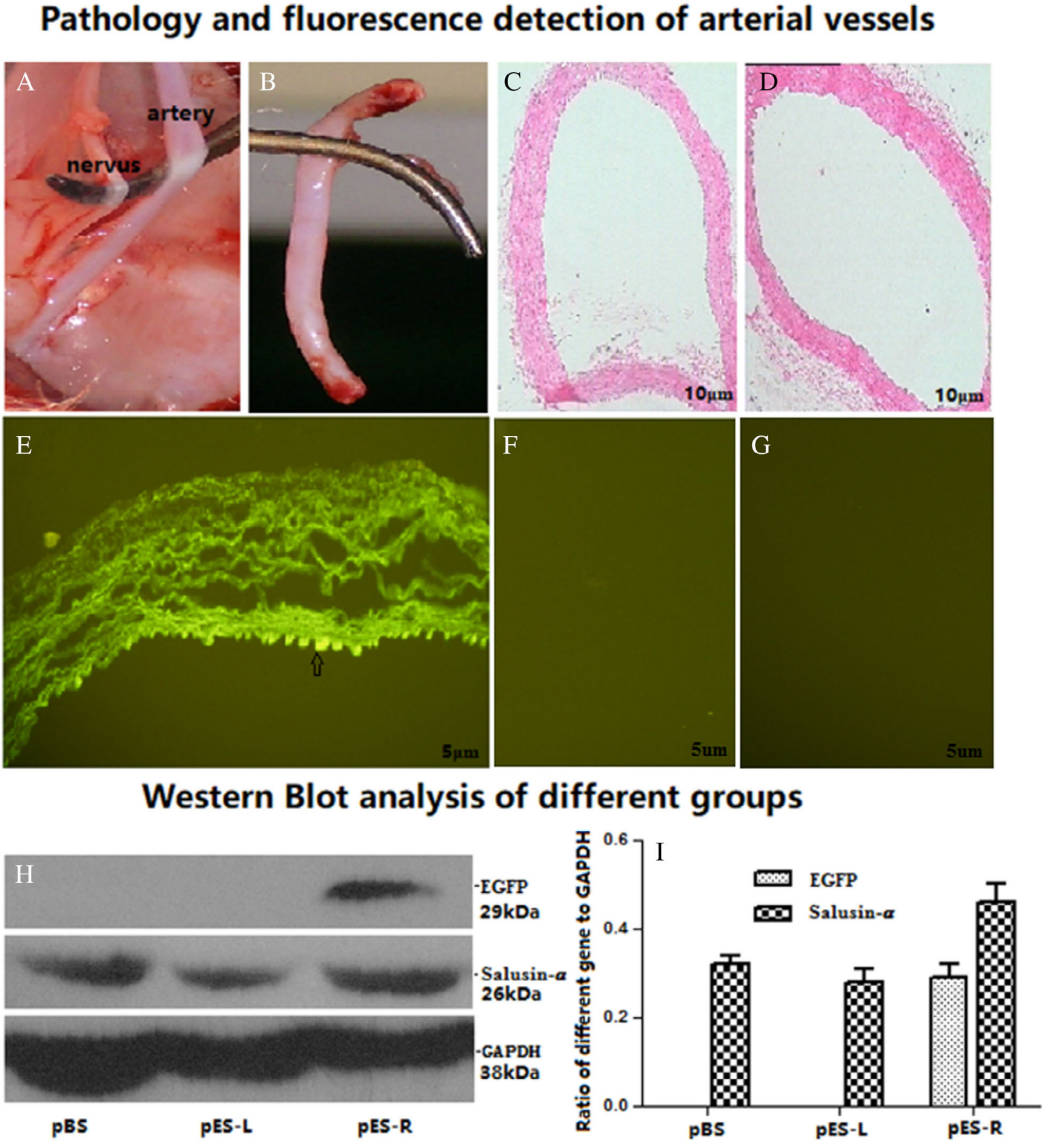
pEGFP‐Salusin‐α transfection of rabbit carotid artery endothelial analysis. **A**, Histopathological examination and fluorescence detection of arterial vessels. Sectioned Arterial vessel before (**A**) and after (**B**) dissection. **C**, A section of a blood vessel transfected with plasmid. **D**, A section of a not‐transfected artery. **E**, Fluorescence detected in arterial endothelium transfected with pEGFP‐Salusin‐α plasmid and **F** and **G** show the absence of fluorescence in arterial endothelium of the controls not transfected with plasmids. The arrow indicates the transfected endothelium. **H**, Western blot analysis of tissue from different groups. Representative western blotting results from the three groups. **I**, The analysis of western blot band densities in the three groups. The data are representative of three independent experiments (*n* = 3).

The common carotid artery and its concomitant nerve were surgically dissected from anesthetized rabbits, which had been previously transfected with pEGFP‐salusin‐α (Figure 4A). The common carotid artery segments that had been transfected with plasmids were obtained (Figure 4B). To check whether targeted transfection had been successful at transferring the plasmid only locally where ultrasonication had been applied, the pES group was divided into two subgroups: pES‐R for the right common carotid artery group and pES‐L for the left common carotid artery group, because the left common carotid artery had not been subjected to ultrasonication and we were not expecting to see any EGFP expression there; while the right common carotid artery had been ultrasonicated with the SonoVue plus plasmid mixture, we were expecting to see EGFP expression there. As shown in Figure 4E for the pES‐R subgroup, we achieved successful transfection of pEGFP‐salusin‐α in endothelial cells of the right common carotid artery endothelium. In fact, the right common carotid arterial endothelium showed green fluorescence, in contrast to the left common carotid arterial endothelium of the pES‐L subgroup shown in Figure 4F and the common carotid arterial endothelium of the rabbits in the pBS group (Figure 4G), which did not show any EGFP expression. Both histological sections of common carotid artery segments, which had been transfected with pEGFP‐salusin‐α plasmid (Figure 4C), and histological sections of common carotid artery segments dissected from rabbits belonging to control groups (pES‐L and pBS), which had not been transfected with the plasmid (Figure 4D), did not show any pathological changes. Gene expression of the transfected plasmid was further detected by western blotting. EGFP gene expression was apparent only in the right common carotid artery endothelium in the pES‐R subgroup (Figure 4H, top). Salusin‐α was expressed in all groups (Figure 4H, middle), which was consistent with Shichiri et al's report that Salusin‐α was widely expressed in various tissues.^19^ Figure 4I shows that Salusin‐α expression was greater in the pES‐R group than in the other groups, and no EGFP protein expression was observed in the pES‐L and pBS groups. These data confirm that the target salusin‐α sequence was efficiently and site‐specifically transfected into vascular endothelial cells, and we observed that its expression was consistent with the results of our previous studies.^18^

### Evaluation of pcDNA3.1 Salusin‐α Transfection of Rabbit Carotid Arterial Endothelium

**Figure 5:**
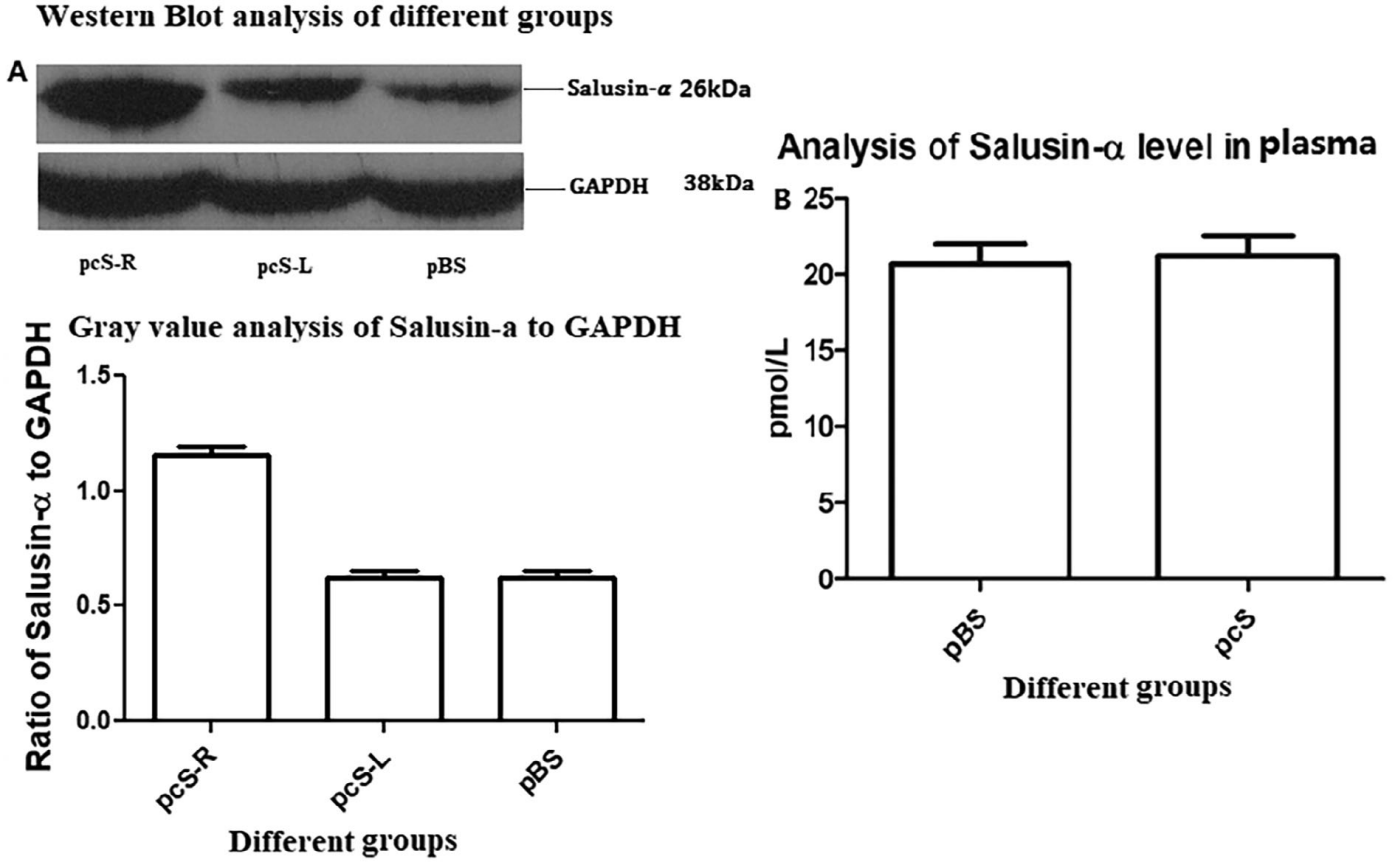
Analysis of pcDNA3.1‐salusin‐α transfection of rabbit carotid artery endothelium. **A**, Western blot analysis of different groups. Representative western blotting results in the three groups (above). Graph (below) showing the analysis of western blot band densities in the three groups. **B**, Analysis of salusin‐α level in plasma. The data are representative of thre independent experiments (*n* = 3).

As for pEGFP‐salusin‐α transfection, also for the transfection of plasmid pCDNA3.1‐Salusin‐α, a control group indicated as pBS group, which was treated with only PBS not containing the plasmid, and an experimental group, which was treated with the pcDNA3.1‐Salusin‐α plasmid indicated as pcS group, were set up. The expression plasmid pcDNA3.1‐Salusin‐α was transfected into the rabbit carotid artery endothelium, and the expression of salusin‐α in each group was analyzed. To analyze the site‐specific transfection of pcDNA3.1‐Salusin‐α, the pcS group was divided into two subgroups: pcS‐R and pcS‐L, representing the right (pcS‐R) and the left (pcS‐L) common carotid artery endothelial tissue. The expression of the salusin‐α peptide in the pcS‐R group was obviously higher than that in the pcS‐L and pBS groups, but there was no significant difference in salusin‐α expression between the pcS‐L and the pBS group (Figure 5A). This confirmed targeted transfection of pcDNA3.1‐Salusin‐α plasmid into the carotid arterial endothelium and showed that the transfected sequence coding for the salusin‐α peptide was expressed correctly.

We performed ELISA to detect plasma salusin‐α levels in pBS and pcS groups (Figure 5B) and found that salusin‐α values were 20.65 ± 2.38 pmol/L for the pBS group and 21.15 ± 2.44 pmol/L for the pcS group, which were not statistically different (*P* = .82). Therefore, transfection of pcDNA3.1‐Salusin‐α into the common carotid arterial endothelium does not seem to affect the plasmatic concentration of salusin‐α. This could be due to the fact that the transfected and overexpressed salusin‐α is not secreted into the blood stream and is retained inside the endothelial cells. This aspect warrants further studies for clarification.

### Evaluation of Rabbit Blood Parameters

Before sacrificing the rabbits, we looked at the differences in various blood parameters of rabbits in the pBS and pcS groups (Table 2). The parameters included were routine blood test parameters such as blood cell counts, liver function, myocardial enzymes, renal function, and blood glucose, and there was no difference in any of the parameters between the two groups (*P* > .05). This indicated that transfection of the plasmid into the rabbit common carotid artery endothelium had no effect on these parameters.

**Table 2:** Blood Parameters in Different Groups

Index	pBS Group	pcS Group	*t*	*P*
WBC (10^9^/L)	8.20 ± 1.11	8.30 ± 0.75	0.54	.64
RBC (10^12^/L)	6.23 ± 0.45	6.20 ± 0.30	0.15	.89
HB (g/L)	129.70 ± 7.77	126.00 ± 9.17	4.16	.15
HCT (%)	41.00 ± 3.00	41.33 ± 1.53	0.15	.89
PLT (10^9^/L)	395.30 ± 48.21	411.30 ± 19.40	0.68	.57
TP (g/L)	54.00 ± 2.00	52.33 ± 5.13	0.59	.62
ALB (g/L)	39.00 ± 2.00	40.33 ± 30.79	0.42	.71
ALT (IU/L)	50.67 ± 2.52	47.67 ± 2.58	1.19	.35
AST (IU/L)	38.67 ± 3.05	39.33 ± 5.86	0.15	.89
CK (IU/L)	84.00 ± 4.58	83.67 ± 2.52	0.18	.87
LDH (IU/L)	63.00 ± 4.58	63.33 ± 7.51	0.06	.96
BUN (mmol/L)	13.70 ± 0.70	12.93 ± 0.35	2.95	.10
CREA (μmol/L)	118.90 ± 5.53	118.40 ± 4.50	0.09	.94
GLU (mmol/L)	6.37 ± 0.31	6.27 ± 0.11	0.87	.48

Variables are presented as mean ± standard deviation. Abbreviations: pBS, group of control rabbits treated with phosphate‐buffered saline; pcS, group of rabbits treated with pcDNA3.1‐Salusin‐α; WBC, white blood cell count; RBC, red blood cell count; HB, hemoglobin; HCT, hematocrit; PLT, blood platelet; TP, total protein; ALB, albumin; ALT, alanine aminotransferase; AST, aspartate aminotransferase; CK, creatine kinase; LDH, lactate dehydrogenase; BUN, blood urea nitrogen; CREA, creatinine; GLU, glucose.

## Discussion

The present study confirmed that the transfected plasmids were delivered into the endothelium of the arterial vessels. In rabbits, the endothelium of the right common carotid artery was successfully transfected with pEGFP‐salusin‐α plasmid, while the endothelium of the left artery, which was used as a control showed no evidence of pEGFP‐salusin‐α plasmid transfection. These results confirmed that the plasmid could be target‐transfected into the artery endothelium. Next, we repeated the same protocol for transfecting vascular endothelial cells with pcDNA3.1‐Salusin‐α expression plasmid, and the results were consistent with those of the previous experiments performed using the pEGFP‐salusin‐α plasmid.^18^ Salusin‐α expression in targeted transfected vascular endothelial tissue was higher than that in other non‐transfected vascular endothelial tissues. Interestingly, no significant difference was observed between the control group and the experimental group regarding the level of plasma salusin‐α, as measured by ELISA. This further supports the feasibility of targeted gene transfer in the vascular endothelium and provides data for clinical targeted gene intervention to combat atherosclerosis.

Atherosclerosis is the result of multiple factors.^20^, ^21^ It is characterized by narrowing of arteries and inadequate blood supply to affected organs.^6^, ^7^ As a consequence, patients show differential symptoms, depending on which organs are affected. With the advancement in technology, it is not difficult to diagnose atherosclerosis clinically; however, there is no effective therapy to treat the disease. Comprehensive therapy is a good intervention for patients with atherosclerosis,^22^, ^23^ but it is a long process that requires great perseverance on the part of the patients. Drug therapy for atherosclerosis is mainly aimed at reducing blood lipids and platelet aggregation, dilating blood vessels, and dissolving thrombi. For this reason, anticoagulant drugs are prescribed for atherosclerosis. Unfortunately, these drugs can also cause many serious adverse effects.^24^, ^25^ These methods also showed inconsistent efficacy.^26^, ^27^, ^28^ Currently, interventions for atherosclerotic stenosis, or occlusion include recanalization, reconstruction, bypass grafting, and endovascular stents.^29^, ^30^ However, even with different drug combinations, it remains difficult to control the re‐narrowing of arteries. Stents with drugs or transfected genes have been reported to inhibit restenosis, but the effect was not significant.^30^, ^31^ Hence, there is an urgent need to discover a novel approach to intervene in steroid‐induced vascular stenosis.

In our previous study,^17^ we showed that SonoVue, an ultrasound contrast agent, has an adhesion rate of approximately 88% to plasmids, and that it can be used to transfect the TFPI gene into the vascular endothelium to inhibit intimal hyperplasia. In 2018, we reported^18^ that transfection of the vasoactive peptide salusin**‐**α coding sequence into the vascular endothelium of rabbits subjected to high‐fat diet induced atherosclerosis effectively inhibited arterial intimal hyperplasia. Our study described a new method for the noninvasive, simple, and efficient transfection of genes into the arterial endothelium. Compared with other methods,^28^, ^29^, ^30^, ^31^ this new method can transfect the same target gene into the endothelium several times as well as at different times. However, whether the transfected gene could be efficiently targeted into the endothelium, whether the transfection method was safe, and whether the level of target gene product in the blood circulation changed had not been evaluated. The vasoactive salusin**‐**α peptide inhibits atherosclerosis^18^ and can be expressed in multiple tissues.^19^ Therefore, we selected salusin**‐**α for transfection into vascular endothelium. First, we cloned salusin‐α coding sequence into both pEGFP‐N1 (Figure 2) and pcDNA3.1 plasmids and compared the adhesion rate of SonoVue to the two new constructs, pEGFP‐salusin‐α and pcDNA3.1‐salusin‐α, with the adhesion rate of SonoVue to pEGFP‐TFPI‐2, which we described previously.^17^ Figure 3 shows that there is no difference in the adhesion rate to SonoVue among different plasmids, a result that has not yet been reported. Interestingly, the different sequences cloned into the plasmids did not appear to affect the adhesion rate of the plasmid constructs to SonoVue. The expression of EGFP and salusin‐α was analyzed by transfection of pEGFP‐salusin‐α plasmid into the arterial endothelium of different groups of rabbits. First, we checked whether the reporter gene (EGFP) or the target vasoactive peptide encoding sequence (salusin‐α) was site‐specifically transfected into the vascular endothelium (Figure 4); this confirmed our previous results.^18^ To further verify the targeted gene delivery, pcDNA3.1‐Salusin‐α expression plasmid was transfected into the arterial endothelium to analyze the expression of salusin‐α in pcS‐R, pcS‐L, and pBS groups. The results showed that salusin‐α expression in pcS‐R group was higher than that in pcS‐L and pBS groups (*P* < .05, Figure 5A). We measured the concentration of salusin‐α in rabbit plasma by performing ELISA and observed no difference between the pcS and pBS groups (*P* > .05, Figure 5B). To evaluate whether transfection of plasmids into the arterial endothelium using SonoVue and ultrasonication is a safe method, we checked multiple blood parameters in the control and experimental groups and observed no difference in any of the parameters between the two groups (*P* > .05, Table 2). These results support the feasibility of targeted delivery of genes into the arterial endothelium.

There are still some open questions arising from the present study that need to be addressed. The plasmids were transfected into the endothelium once a week, for 2 weeks. We do not know how long the expression of constructs transfected into the endothelium can potentially last, and additional research will have to be carried out to answer this question. Another point that will require further investigation is whether different target genes could be transfected efficiently at different times and still elicit expression, or whether two target genes could be transfected at the same time. We looked for expression of transfected constructs in the rabbit right common carotid artery (experimental group) and the left common carotid artery (control group), but we did not look for expression in other tissues, therefore we cannot exclude it *a priori*. Although no rabbit died during the course of the experiments, a general healthy condition of the rabbits could only be confirmed through the analysis of biochemical parameters provided by a routine blood test, which cannot exclude other underlying health conditions. The mechanism of transfection of target genes into the arterial endothelium is not clear. Also, the mechanism by which plasmids adhere to SonoVue is not yet understood. How plasmids adhere to SonoVue in the arterial blood flow is another open question that will require further research to be fully answered.

In conclusion, we established a targeted, noninvasive, simple, safe, and efficient method for transfecting target genes into the common carotid arterial endothelium. Although further research is needed, the results of this study provide data for clinical intervention in atherosclerosis and new ideas for clinical gene therapy.
